# Effects of Synergistic Regulation of Functional Fertilisers and Vermicompost on Soil Fertility and the Growth and Quality of Two Tomato Varieties

**DOI:** 10.3390/plants14203224

**Published:** 2025-10-20

**Authors:** Tianmi Zhang, Kangjie Zhang, Wenhao Zhang, Xuefeng Zhang, Mengyao Cheng, Ruilong Bao, Mingke Zhang

**Affiliations:** College of Horticulture, Northwest Agriculture and Forestry University, Yangling District, Xianyang 712100, China; tianmizhang2024@163.com (T.Z.); zhangkangjie@nwsuaf.edu.cn (K.Z.); z15239088920@outlook.com (W.Z.); zhangxuefeng@nwafu.edu.cn (X.Z.); dimple1932@outlook.com (M.C.); q155580@outlook.com (R.B.)

**Keywords:** functional fertilisers, vermicomposting, tomato quality, soil fertility, synergistic effects

## Abstract

The quality of tomato fruit represents a key determinant of consumer preference, while functional fertilisers significantly contribute to quality enhancement. Limited research has investigated the synergistic mechanisms between functional fertilisers and vermicompost in tomato cultivation systems. The present study was designed to investigate the effects of synergistic regulation between functional fertilisers and vermicompost on soil fertility, as well as the growth and quality of two tomato cultivars, with the ultimate goal of identifying the functional fertiliser treatment exhibiting optimal comprehensive performance. A completely randomised block design was adopted, involving two tomato cultivars (DRK0568 and Sangfen 180), five functional fertiliser treatments (T1–T5), and a water-only control (CK). Measurements included tomato growth parameters, photosynthetic characteristics, fruit quality indices, yield components, biomass accumulation, soil nutrient levels, and enzyme activities. The results demonstrated significant varietal-specific responses to different functional fertiliser treatments. In terms of growth and yield, the T1 treatment exhibited a significant advantage, as it significantly increased the plant height, stem thickness, biomass, and yield of both varieties (DRK0568 and Sangfen 180) by 6.86% and 10.41%, respectively, while also significantly reducing the malformed fruit rate. For photosynthetic analyses, the T1 treatment significantly increased the chlorophyll a and total chlorophyll content in Sangfen 180, as well as the transpiration rate of both tomato varieties. The T4 treatment resulted in the highest chlorophyll b content and optimal water use efficiency in Sangfen 180. Regarding nutritional quality, the T1 treatment significantly increased the vitamin C and soluble sugar content in DRK0568; both varieties exhibited higher sugar–acid ratios under the T3 and T4 treatments. A comprehensive evaluation using the entropy-weighted TOPSIS method for multiple quality indicators (excluding yield parameters) showed that the T4 treatment achieved the highest score. Soil nutrient analyses revealed that the T1 treatment significantly increased the soil organic matter and available potassium content in DRK0568, while the T4 treatment significantly increased the urease activity in Sangfen 180. In conclusion, the T1 treatment (mineral-sourced potassium fulvate fertiliser) exhibited excellent performance in both increasing yield and improving quality, while the T4 treatment (Type II algal polysaccharide fertiliser additive) demonstrated unique advantages in enhancing fruit quality indicators.

## 1. Introduction

Tomato fruit is a valuable source of minerals and vitamins necessary in the human diet [1]. Amid increasing consumer awareness of food safety and nutritional health, the demand for high-quality tomatoes is on the rise [2,3,4]. The comprehensive flavour and nutritional characteristics of the fruit, as crucial quality indices, significantly influence consumer preferences and are vital determinants of purchasing intentions [5,6]. Tomato quality represents a multifaceted trait, encompassing diverse processes at both the plant and fruit levels that are governed by the interactions of cultural practices, genetic factors, and environmental conditions [7].

In agricultural science and fertiliser research, functional fertilisers are a new class of fertilisers that supply basic nutrients, integrate functional components, and exhibit targeted functions—specifically, improving soil microecology, enhancing crop stress tolerance, optimising agricultural product quality, and regulating efficient soil nutrient utilization. Functional fertilisers can provide nutrients needed for plant growth and regulate plant physiological and metabolic processes, so they have gradually attracted the attention of researchers [8,9,10,11]. Ochoa-Velasco et al. [12] found that bacillus licheniformis has a positive effect on the content of total flavonoids and antioxidant activity and can regulate the synthesis of antioxidant compounds in plants. The use of organic fertilisers not only improves the yield but also significantly enhances the quality of agricultural produce by increasing desirable traits such as soluble solids, lycopene, and vitamin C while managing nitrate content effectively [13]. Bacterial fertiliser treatments significantly affect plant growth parameters and fruit mineral content, enhancing both yield and quality [14]. Azomite supplementation, which increases tomato yield, subtly but significantly alters the plant-associated microbial community [15]. The studies mentioned above show that functional fertilisers are widely used in tomato cultivation. The application of functional fertilisers can improve tomato quality, enhance fruit flavour, and improve soil environment.

Soil organisms are widely recognised as having the potential to regulate soil fertility and facilitate plant growth. Interactions between plants and soil organisms are essential for nutrient uptake by plants [16]. Bhadauria and Ramakrishnan [17] found that earthworms participated in the N cycle through worm cast egestion, mucus production, and dead tissue decomposition. Vermicomposting involves a joint decomposition process by earthworms and micro-organisms, and is recognised as an excellent product rich in plant nutrients and phytohormones [18,19,20]. Vermicomposting is an agricultural system that integrates vermiculture and vegetable cultivation. This system addresses the issue of environmental pollution caused by vegetable waste through recycling agricultural waste, utilising vermicompost—produced by earthworms processing organic waste—as a substrate or fertiliser for tomato cultivation, while also enabling in situ resource utilization and soil quality conservation.

Currently, most studies have focused on the effects of vermicompost and functional fertilisers when applied alone, with a lack of in-depth analysis of the intrinsic mechanism underlying their synergistic effects. This is particularly true for key aspects such as the interaction between beneficial microorganisms in vermicompost and specific components of functional fertilisers, the impact of vermicompost on nutrient release and bioavailability in functional fertilisers, and how the two synergistically regulate tomato quality formation, which remain unclear. An in-depth investigation into this synergistic mechanism can not only offer novel insights and approaches for the precise regulation of tomato quality, but also lay a theoretical foundation for the rational application of functional fertilisers.

In this study, using a vermicompost trough cultivation system, we systematically analysed two tomato varieties with respect to their growth and development, physiological traits, yield, quality, and changes in soil nutrient elements through the establishment of distinct functional fertiliser treatment groups. This study aimed to evaluate the combined efficacy of functional fertilisers and vermicompost in improving soil fertility and enhancing the growth and quality of two tomato cultivars. Furthermore, it sought to elucidate the mechanisms through which functional fertilisers operate within a vermicompost-based system and to identify the optimal functional fertiliser treatment based on overall performance.

## 2. Results

### 2.1. The Effects of Five Functional Fertiliser Treatments on Tomato Growth

**Figure 1:**
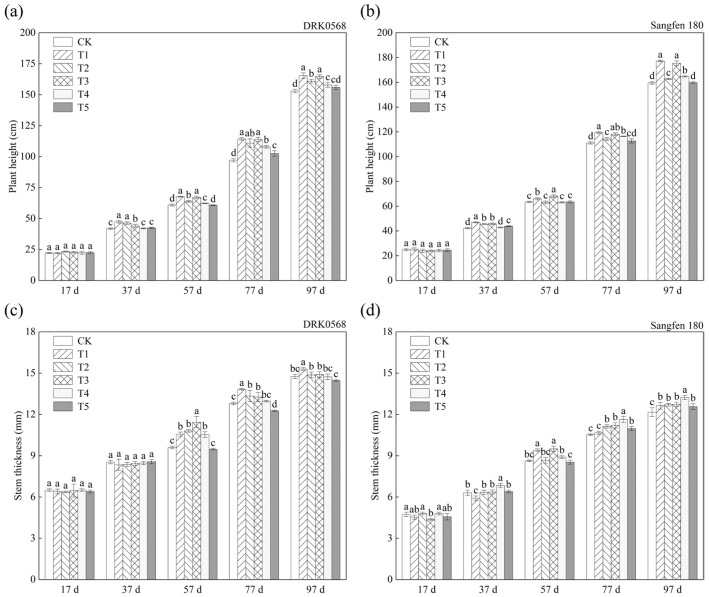
Effects of different functional fertiliser treatments on tomato growth. (**a**) DRK0568 variety plant height; (**b**) Sangfen 180 variety plant height; (**c**) DRK0568 variety stem thickness; (**d**) Sangfen 180 variety stem thickness. Plant height and stem diameter of the two tomato varieties were measured at 17, 37, 57, 77, and 97 days after transplantation. Different lowercase letters indicate significant differences in the level of the same condition under different treatments (*p* < 0.05).

The plant height of the DRK0568 variety was significantly higher in all treatment groups (T1 > T3 > T2 > T4 > T5) than in CK. It reached its maximum at 97 days after transplanting, with respective increases of 8.17%, 7.65%, 5.23%, and 3.2% compared to CK (Figure 1a). The T5 treatment exhibited a greater plant height than CK at 57 days after transplanting and showed a 1.91% increase compared to CK at 97 days after transplanting (Figure 1a). Stem thickness increased during the reproductive stage. The stem thickness of the T1, T2, and T3 treatments exceeded that of CK at 37 days after transplanting (T1 > T3 > T2) and reached the maximum at 97 days after transplanting, with respective increases of 3.61%, 0.9%, and 0.67% compared to CK. In contrast, the stem thickness of the T4 and T5 treatments was lower than that of CK (Figure 1c).

Plant height in all treatments of Sangfen 180 showed an increasing trend from 17 to 97 days after transplanting (Figure 1b). During 17 to 97 days after transplanting, the plant height of the T1 and T3 treatments was significantly higher than that of the other treatments, reaching 177.2 cm and 175.4 cm at 97 days after transplanting, with respective increases of 11.0% and 9.9% compared to CK (Figure 1b). Significant differences in stem thickness were observed in Sangfen 180 from 17 to 97 days after transplanting. The maximum stem thickness (13.2 mm) was recorded in the T4 treatment at 97 days after transplanting, which was 8.2% higher than that of CK. All other treatments also exhibited a higher stem thickness than CK (Figure 1d).

### 2.2. Effects of Five Functional Fertiliser Treatments on Photosynthetic Parameters of Two Tomato Cultivars

**Figure 2:**
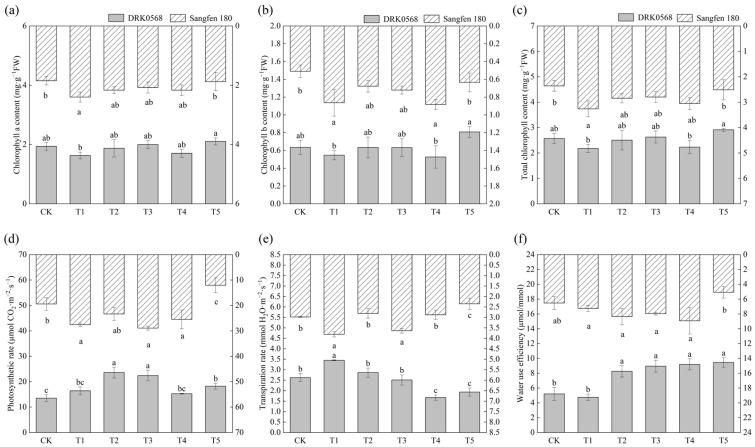
Effects of different functional fertilisers on the photosynthetic parameters of tomato. (**a**) Chlorophyll a content, (**b**) chlorophyll b content, (**c**) total chlorophyll content, (**d**) photosynthetic rate, (**e**) transpiration rate, and (**f**) water use efficiency. Different lowercase letters indicate significant differences in the level of the same condition under different treatments (*p* < 0.05, *n* = 3).

As shown in Figure 2, the chlorophyll a content in the T1 treatment of Sangfen 180 was significantly higher than that in the other treatments (Figure 2a); the T4 treatment of Sangfen 180 exhibited the highest chlorophyll b content (Figure 2b); and the T5 treatment of DRK0568 had higher chlorophyll a and chlorophyll b content than the other treatments (Figure 2a,b). Taken together, the T1 treatment resulted in the highest total chlorophyll content in Sangfen 180, while the T5 treatment led to the highest total chlorophyll content in DRK0568, with respective increases of 38.14% and 13.23% compared to CK (Figure 2c). Except for the T5 treatment of Sangfen 180, the photosynthetic rates of the two tomato varieties under the remaining treatments were all higher than those of CK (Figure 2d). The highest photosynthetic rates of DRK0568 and Sangfen 180 were observed in the T2 and T3 treatments, which were 74.21% and 48.74% higher than those in CK, respectively. Under the T1 treatment, the transpiration rates of DRK0568 and Sangfen 180 were significantly higher than those in the other treatments, being 31.68% and 27.85% higher than those in CK, respectively (Figure 2e). In terms of water use efficiency, DRK0568 achieved the highest water use efficiency under the T5 treatment, while Sangfen 180 reached the highest water use efficiency under the T4 treatment. Compared with the CK group, both varieties showed an increase in water use efficiency under their respective optimal treatments (Figure 2f).

### 2.3. Effects of Five Functional Fertiliser Treatments on Tomato Quality Traits

**Figure 3:**
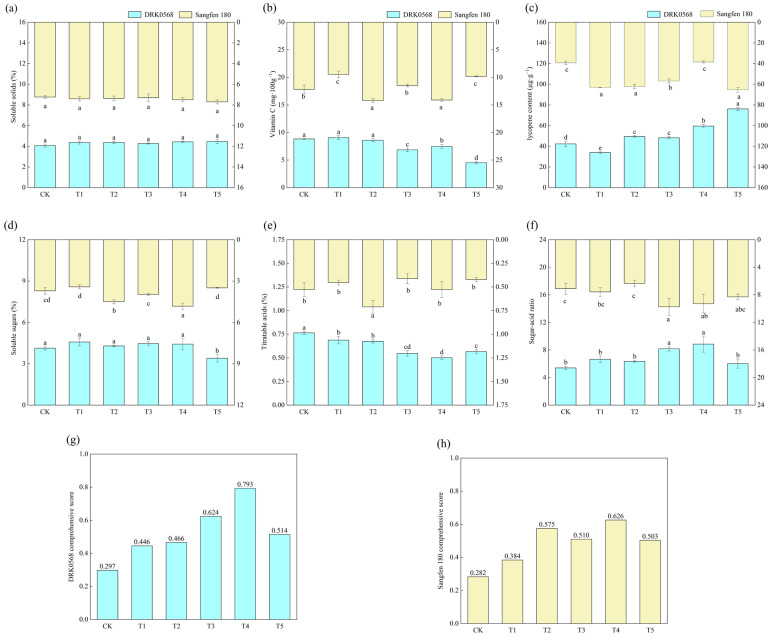
Effects of different functional fertilisers on the nutritional quality of tomato. (**a**) Soluble solids, (**b**) vitamin C, (**c**) lycopene content, (**d**) soluble sugars, (**e**) titratable acids, (**f**) sugar–acid ratio, (**g**) DRK0568 comprehensive score, and (**h**) Sangfen 180 comprehensive score. Different lowercase letters indicate significant differences in the level of the same condition under different treatments (*p* < 0.05, *n* = 3).

Soluble solid content did not differ significantly among treatments; all values exceeded those in CK, with Sangfen 180 consistently demonstrating higher values than DRK0568 (Figure 3a). DRK0568 reached peak vitamin C and soluble sugar content under the T1 treatment, whereas Sangfen 180 showed minimal values for these parameters (Figure 3b,d). Lycopene content in both varieties peaked significantly under the T5 treatment, showing an 80.03% increase relative to CK (Figure 3c). DRK0568 exhibited the maximum titratable acid content in CK and the minimum in T4, whereas Sangfen 180 showed the highest and lowest levels in T2 and T3, respectively (Figure 3e). The sugar–acid ratios differed significantly between treatments, with T3 and T4 yielding consistently higher values in both varieties compared to the other treatments (Figure 3f). Through the entropy-weighted TOPSIS model for comprehensive nutritional quality evaluation, both DRK0568 and Sangfen 180 varieties achieved the highest comprehensive nutritional quality scores under the T4 treatment, with values of 0.79 and 0.63, respectively (Figure 3g,h).

### 2.4. Effects of Five Functional Fertiliser Treatments on Tomato Yield and Malformed Fruit Rate

**Figure 4:**
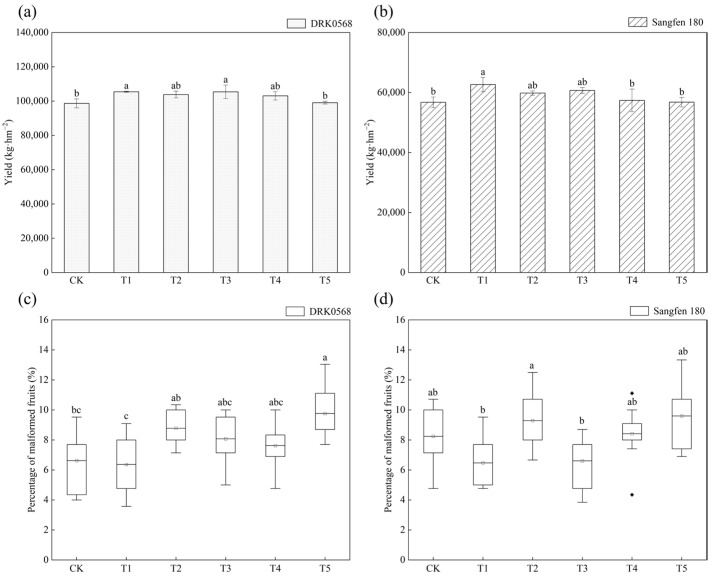
Effect of five functional fertilisers on the yield and malformed fruit rate of two tomato varieties. (**a**) DRK0568 yield; (**b**) Sangfen 180 yield; (**c**) DRK0568 malformed fruit rate; (**d**) Sangfen 180 malformed fruit rate. Different lowercase letters indicate significant differences in the level of the same condition under different treatments (*p* < 0.05).

The treatments exerted significant effects on both yield and malformed fruit rate in both DRK0568 and Sangfen 180 varieties (Figure 4). Regarding yield, DRK0568 showed significant increases under T1 (6.86%) and T3 (6.83%) compared to CK, while T2, T4, and T5 were not statistically different from CK (Figure 4a). By contrast, only T1 increased yield significantly (10.41%) in Sangfen 180, while the other treatments did not differ significantly from CK (Figure 4b). With respect to the malformed fruit rate, the T5 treatment in DRK0568 was significantly higher than that in both the CK and T1 treatments, representing a 47.53% increase compared with the CK group (Figure 4c). In Sangfen 180, T2 and T5 treatments had significantly higher malformed fruit rates than T1 and T3, but no difference comparable to CK, whereas T1 and T3, though numerically lower than CK, did not differ significantly (Figure 4d). Collectively, cultivar responses differed by applying five functional fertilisers: T1 increased yield while decreasing the malformed fruit rate in both varieties; T3 improved yield only in DRK0568. While the T2, T4, and T5 treatments exhibited minimal effects on yield, the T2 and T5 treatments significantly increased malformed fruit rates.

### 2.5. Effects of Five Functional Fertiliser Treatments on Tomato Biomass

**Figure 5:**
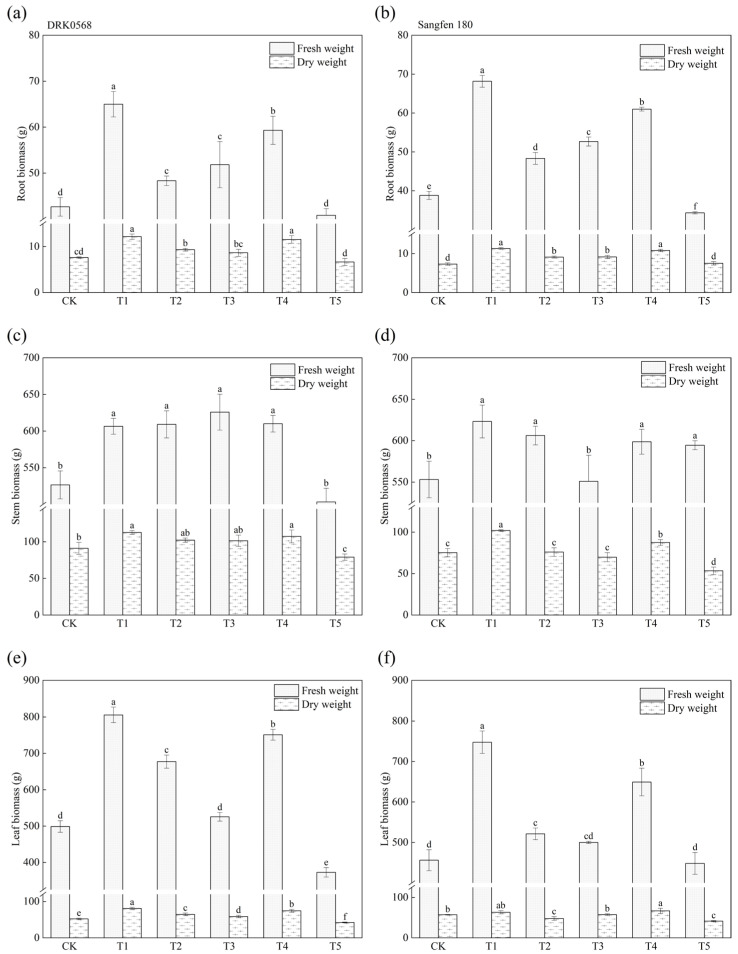
Effect of five functional fertilisers on the biomass of two tomato varieties. (**a**) DRK0568 root biomass, (**b**) Sangfen 180 root biomass, (**c**) DRK0568 stem biomass, (**d**) Sangfen 180 stem biomass, (**e**) DRK0568 leaf biomass, and (**f**) Sangfen 180 leaf biomass. Different lowercase letters indicate significant differences in the level of the same condition under different treatments (*p* < 0.05, *n* = 3).

The trends in tomato root biomass changes were consistent across treatments, except for T5. Both fresh and dry root weights in the T1 to T4 treatments were significantly higher than those in CK, with T1 showing the most pronounced effects (Figure 5a,b). Compared to CK, DRK0568 showed 52.34% and 59.39% increases in fresh and dry root weights, respectively, while Sangfen 180 exhibited 76.52% and 54.54% increases (Figure 5a,b). Regarding stem biomass, both varieties showed significantly higher fresh weights in T1, T2, and T4 treatments; dry weights were most elevated in T1 and T4, with DRK0568 increasing by 23.8% and 17.95%, and Sangfen 180 by 35.39% and 16.15% relative to CK (Figure 5c,d). DRK0568 exhibited significantly greater leaf biomass in T1 and T4. For Sangfen 180, leaf fresh weight increased by 63.96% (T1) and 42.39% (T4) relative to CK, while dry weight was highest in T4 among all treatments (Figure 5e,f).

### 2.6. Effects of Five Functional Fertiliser Treatments on Soil Fertility

#### 2.6.1. Soil Physicochemical Properties

Experimental data revealed that the five functional fertiliser treatments exerted significant differential effects on the contents of soil organic matter (SOM), soil pH, total nitrogen (TN), total phosphorus (TP), total potassium (TK), alkali-hydrolyzable nitrogen (AN), available phosphorus (AP), and available potassium (AK) in the rhizosphere soil of tomato cultivars DRK0568 and Sangfen 180 (Table 1). Moreover, these effects exhibited cultivar-specific variations. Distinct cultivar-specific responses in soil organic matter (SOM) dynamics were observed: DRK0568 exhibited significantly higher SOM content under T1 treatment compared to both the CK and T4 treatments, whereas Sangfen 180 demonstrated superior SOM accumulation, specifically under the T3 treatment, significantly surpassing all other treatments. Regarding rhizosphere pH profiles, DRK0568 consistently maintained lower values than Sangfen 180 across T1, T2, T3, and T4 functional fertiliser applications; conversely, under the T5 treatment, the pH of DRK0568 (8.09) was greater than that of Sangfen 180 (8.01). Concurrently, both cultivars attained maximum TP and TK concentrations during the T5 treatment, with DRK0568 showing significantly higher TP and TK levels versus the T3 treatment, though no statistically significant differences emerged relative to the other treatments. Notably for DRK0568 under T2, TN, AN, and AP contents were significantly elevated versus CK, while AK content was markedly reduced. Additionally, its T1 treatment yielded significantly higher AK content than all other treatments. Conversely, Sangfen 180 under T3 displayed significantly greater SOM and TN contents compared to all treatments, with AP content significantly exceeding T4, but showing no statistical difference with the T5 treatment.

#### 2.6.2. Soil Enzyme Activity

The application of five functional fertilisers elicited significant variations in soil enzyme activity (Table 2). The T2 treatment induced a 62.37% elevation in urease activity in DRK0568, whereas the T4 treatment resulted in a 61.40% increase in urease activity in Sangfen 180, both compared to CK. Under the T3 treatment, catalase activity was elevated by 24.04% and 35.25% in DRK0568 and Sangfen 180, respectively, relative to CK. The T1 and T5 treatments significantly increased sucrase and alkaline phosphatase activities in both tomato varieties compared to CK.

## 3. Discussion

### 3.1. Differential Regulation of Tomato Growth Physiology and Yield by Functional Fertilisers

The results of this study demonstrated that different functional fertilisers exerted differential effects on tomato growth and biomass. All treatments, except T5, significantly increased tomato plant height, with the T1 treatment exhibiting the most pronounced effect (Figure 1a). This observation is consistent with previous findings regarding plant growth promotion by organic fertilisers [21]. Biomass analysis further confirmed that the T1 treatment significantly increased the fresh and dry weights of the roots, stems, and leaves (Figure 5). Specifically, the root biomass of Sangfen 180 increased by up to 76.52%, which reflects the mechanism by which functional fertilisers promote root development [22]. However, under the T4 and T5 treatments, stem thickness was significantly lower than that in the CK group (Figure 1b,d), which may be associated with the inhibition of lignification induced by high nitrogen supply [23]. The inhibitory effect of T5 on stem thickness and biomass is inconsistent with its superiority in photosynthetic parameters, which is presumed to result from a preferential shift in carbon allocation toward leaves rather than stems.

Photosynthesis in leaves and fruits coordinately promotes tomato growth and development [24]. The photosynthetic parameter results (Figure 2) indicated that the T5 treatment resulted in the highest water use efficiency (WUE) in DRK0568, whereas the lowest WUE was observed in Sangfen 180. This phenomenon may be associated with the distinct stomatal regulation strategies among varieties: DRK0568 may have adapted to the T5 treatment by reducing transpiration rates, whereas Sangfen 180 maintained high transpiration rates, resulting in water loss. Additionally, differences in stomatal density directly affect stomatal conductance (Gs) and transpiration, thereby influencing the calculation results of the WUE based on Pn/Tr.

Yield analysis (Figure 4a,b) revealed that the T1 treatment increased yields in both varieties, which is consistent with the observed increases in biomass and photosynthetic efficiency. However, the T3 treatment was effective only for DRK0568, indicating the presence of a threshold in varietal yield responses to fertilization. The deformed fruit rate was significantly higher under the T5 treatment, which may be associated with fertiliser-induced hormonal imbalance or abiotic stress (Figure 4c,d). The T1 treatment increased yield while reducing the deformed fruit rate, whereas the T3 treatment increased yield but did not significantly improve fruit quality. It is hypothesised that the T1 treatment achieves high quality and high yield by balancing nutritional and hormonal signals, whereas the T3 treatment may only promote vegetative growth. Furthermore, the T4 treatment exhibited no significant effect on yield; however, it showed superior performance in biomass accumulation and water use efficiency (WUE), indicating a greater contribution to plant vigour than fruit development. These findings provide novel insights into the functional positioning of fertilisers.

### 3.2. Differences in Tomato Nutritional Indicators in Response to Functional Fertilisers 

In this study, we found that two tomato varieties exhibited significant differences in their response to functional fertilisers, primarily in terms of lycopene content, sugar-acid ratio, and overall score (Figure 3). Among these varieties, the lycopene content of both reached its highest level under the T5 treatment, which is consistent with the previous finding [25,26,27] that specific nutrient or stress treatments can promote lycopene synthesis. However, the trends of other quality indicators under the treatments did not coincide with those of lycopene, suggesting that lycopene synthesis may be governed by independent regulatory pathways that differ from the regulatory mechanisms of sugar and acid metabolism. In addition, no significant difference in soluble solids content was observed between treatments in this study, which is inconsistent with previous findings [28,29] indicating that organic additives or foliar sprays can significantly increase this index. This discrepancy may be attributed to varietal characteristics or differences in treatment composition.

The genetic background of tomato varieties significantly affects nutrient accumulation; for example, Sangfen 180 consistently exhibited higher soluble solids content than DRK0568 (Figure 3a). There was a significant interaction between genotypes and treatments: in DRK0568, the content of soluble solids and soluble sugars peaked under the T1 treatment, whereas in Sangfen 180, these indicators were significantly lower. In addition, the T3 and T4 treatments significantly increased the sugar–acid ratio and composite score, which is consistent with the finding that balanced nutritional supplementation improves fruit flavour quality [30,31,32]. Both tomato varieties achieved high composite scores under the T4 treatment, indicating that this treatment optimally balanced the biochemical properties and organoleptic quality of the fruits. However, its underlying mechanism of action remains to be further investigated.

The titratable acid content peaked in the CK of DRK0568, whereas it peaked in the T2 treatment of Sangfen 180 (Figure 3e). This difference may reflect variations in the regulatory mechanisms of organic acid metabolism among varieties or differences in their ability to adapt to environmental and nutrient signals. Meanwhile, the pattern of titratable acid changes was inconsistent across varieties under different treatments, which may be associated with confounding factors such as variations in rhizosphere microbial activity and differences in treatment uptake efficiency. Under the T4 treatment, DRK0568 exhibited a higher composite score despite its lower titratable acid content, indicating that titratable acid had a lower weight in the composite evaluation (Figure 3g,h). Meanwhile, consumers prefer sweetness over acidity [33].

### 3.3. Soil Nutrient Transformation and Enzyme Activity Response Regulated by Fertiliser Type

The present study revealed species-specific effects of fertiliser type on soil nutrients and pH (Table 1). The T1 and T3 treatments increased soil organic matter content, which may be associated with the transformation of soil organic matter by heterotrophic microbial communities: heterotrophic microbial communities are key drivers of SOM transformation. Different microorganisms metabolise various compounds in plant residues at varying rates; for complex molecules, decomposition requires specific microorganisms and their secreted extracellular enzymes. Furthermore, during decomposition, the microbial community undergoes corresponding changes as SOM composition alters [34,35]. In addition, the T5 treatment maximised the total phosphorus (TP) and total potassium (TK) contents in both tomato varieties. Notably, although soil pH was generally lower in DRK0568 than in Sangfen 180, it was greater in DRK0568 than in Sangfen 180 under the T5 treatment. This may be attributed to the fertiliser containing phosphorus and potassium components: phosphorus, with low mobility, tends to accumulate in the soil [36], and crops have low potassium uptake [37]. Consequently, the application of this fertiliser increased both TP and TK contents, whereas other fertilisers either lacked these components or exhibited more pronounced depletion due to absorption. Plant growth and physiological functions are supported by the nutrient chemistry of essential N, P, and K fertiliser elements [38]. Meanwhile, soil adsorption and desorption of phosphorus are influenced by pH. Lowering pH alters the soil surface charge properties, reduces soil phosphorus adsorption, promotes phosphorus desorption from the soil solid phase into the soil solution, and increases phosphorus availability [39].

Soil enzyme activities are involved in various soil biochemical processes [40]. The selection of enzymes for soil quality assessment is grounded in three key factors: their responsiveness to soil management practices, their involvement in organic matter decomposition, and the comparative simplicity of their analytical procedures [41]. Soil enzymes exhibit operational practicality and integrative properties, are simple to measure, and importantly, they respond to changes in soil management much earlier than other detectable shifts in soil quality indicators [42]. According to Bharti et al. [43], fertilisers regulate the types and quantities of secreted enzymes by altering the species, abundance and metabolic environment of microorganisms, which in turn affects enzyme activity.

The changes in soil enzyme activities observed in this study are attributed to the combined effects of the native soil microbial community and any enzymes potentially introduced with exogenous organic amendments. Notably, since all treatments were applied on an equal basis of vermicompost, the differences observed are likely primarily driven by the sustained priming effect exerted by the functional fertilisers on the microbial community, rather than a mere addition of exogenous enzymes. It was found that the T2 and T4 treatments increased urease activity by more than 60% in the soil of planting both tomato varieties, the T3 treatment significantly enhanced catalase activity, and the T1 and T5 treatments generally increased sucrase and acid phosphatase activity (Table 2). This phenomenon can likely be attributed to the unique active constituents (e.g., humic acids, seaweed polysaccharides, fish protein peptides, and silicon) present in the different functional fertilisers [44,45]. These constituents likely function as high-quality metabolic substrates or signalling molecules, which more persistently modulate the structure and functional activity of the soil microbial community, including its capacity for enzyme synthesis and secretion. Consequently, these processes mediate the distinct differences in enzyme activities observed across treatments.

## 4. Materials and Methods

### 4.1. Experimental Conditions

**Figure 6:**
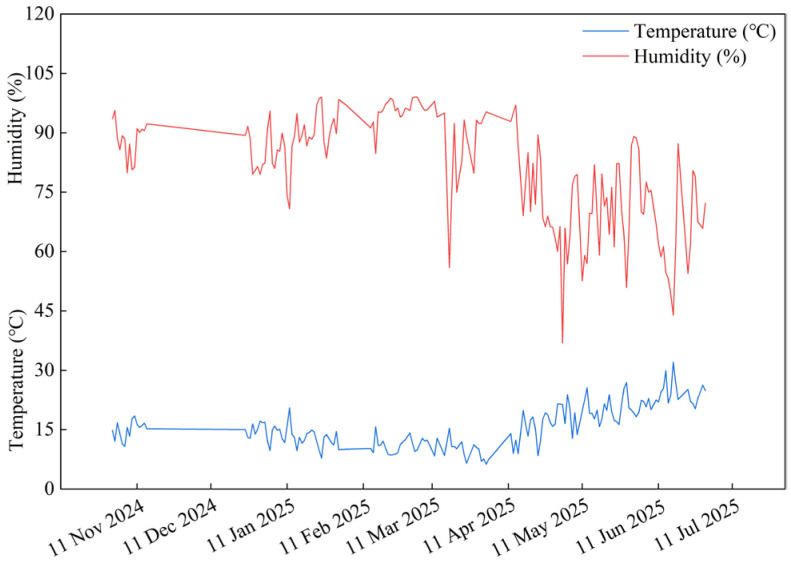
Greenhouse meteorological data during the experiment.

The experiment was conducted in a solar-heated greenhouse at the Jingyang Vegetable Experimental Station (34°35′40″ N, 108°49′9″ E), Xianyang City, Shaanxi Province, China, between September 2024 and June 2025. The site elevation is 474.6 m above sea level, with a warm temperate continental monsoon climate (mean annual temperature: 13 °C; precipitation: 548.7 mm). The meteorological data in the experimental solar greenhouse are shown in Figure 6. The average nutrient content of the soil layer from 0 to 20 cm before transplantation was organic matter 43.36 g·kg^−1^, total nitrogen 2.22 g·kg^−1^, total phosphorus 1.81 g·kg^−1^, total potassium 22.52 g·kg^−1^, available nitrogen 379.13 mg·kg^−1^, available phosphorus 86.58 mg·kg^−1^, available potassium 313.00 mg·kg^−1^, and the soil pH value was 7.58.

### 4.2. Experimental Materials

The tomato varieties used in the trial were DRK0568 (Netherlands) and Sangfen 180 (China). DRK0568 is an indeterminate, large-fruited tomato variety with strong growth vigour, no leaf yellowing, no premature senescence, and high yield. The fruit has pink skin, a regular shape, high firmness, good transportability, a single fruit weight of 250–300 g, and high disease resistance. Sangfen 180 is a flavourful indeterminate tomato variety with pink fruit, green shoulders, a highly oblate shape, a single fruit weight of approximately 80–120 g, and resistance to the tomato yellow leaf curl virus.

The functional fertilisers are as follows: T1: Mineral-sourced potassium fulvate fertiliser (humic substance ≥ 55% (fulvic acid ≥ 50% thereof), K_2_O ≥ 12%); T2: Humic acid water-soluble fertiliser (NPK ≥ 20%, potassium fulvic acid ≥ 20%, humic acid ≥ 4%, seaweed extract ≥ 2%); T3: Water-soluble NPK fertiliser with silicon (N:110 g/L, P_2_O_5_:170 g/L, K_2_O:220 g/L, B:2 g/L, Zn:1 g/L); T4: Type II algal polysaccharide fertiliser additive (alginic acid ≥ 20 g/L); T5: Peptide-enriched fish protein organic liquid fertiliser (fish protein ≥ 138 g/L, organic matter ≥ 121 g/L, amino acid ≥ 19 g/L, humic substance ≥ 112 g/L, fulvic acid ≥ 93 g/L).

### 4.3. Experimental Design and Treatments

**Figure 7:**
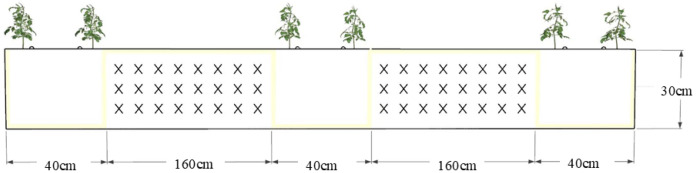
Tomato cultivation mode.

Vermicompost was applied at 20 kg per plot before tomato planting. The experiment employed a randomised complete block design with three replicates, featuring protective border rows. Each plot contained 40 tomato plants arranged with 27 cm intra-row spacing, 40 cm inter-row spacing, and 160 cm main alleyways (Figure 7).

The field experiment comprised five treatments, including a water-only control. Functional fertiliser applications were administered monthly via drip irrigation from November 2024 through April 2025. Fertiliser application rates followed manufacturer specifications (Table 3), with all treatments delivered through a pressurised drip irrigation system following complete dissolution in irrigation water. An integrated water–fertiliser management regime was implemented, with additional agronomic practices following conventional tomato production protocols for the region.

### 4.4. Data Collection

#### 4.4.1. Plant Growth and Fruit Yield

From 9 November 2024, plant height and stem diameter were recorded at 20-day intervals for all labelled plants across treatments. Tomato harvesting commenced on 21 January 2025 and concluded on 9 May 2025 upon plant termination. Total yield was recorded for each treatment plot and converted to yield per hectare based on plot area. Nine plants per treatment were randomly selected, permanently labelled, and evaluated for total fruit yield and deformed fruit count to determine the percentage of fruit deformation. At the end of the harvest, intact plant samples were collected. The roots were carefully rinsed to remove adhering soil and gently blotted dry. Fresh biomass of both the aboveground parts and root systems was immediately determined. The samples were oven-dried in batches, first at 105 °C for 60 min to inactivate enzymes, then at 75 °C until a constant weight was achieved. The dry weights of the aboveground parts and root systems were recorded separately.

#### 4.4.2. Photosynthetic Parameters

Net photosynthetic rate, transpiration rate, and stomatal conductance of tomato functional leaves were determined using a portable porometer (LI-1600 model, LI-COR, Lincoln, NE, USA) from 8:00 to 10:00 a.m. on sunny days selected for the fruiting season [46]. Water use efficiency (WUE) was calculated as the ratio of the net photosynthetic rate (Pn) to the transpiration rate (Tr). Chlorophyll content was quantified spectrophotometrically following Wellburn’s extraction protocol [47].

#### 4.4.3. Quality Indicators

At the peak fruiting stage, five representative plants per plot were randomly selected. From each plant, five fruits of uniform size and maturity (based on the visual colour index) were collected and immediately transported on ice to the laboratory for nutritional quality analysis. A portable digital refractometer (PR-101 model, ATAGO, Tokyo, Japan) was used to measure the TSS content of tomato fruit juice [48]. Titratable acid content was determined by acid–base titration [49]; lycopene content was determined by spectrophotometry [50]. Soluble sugar content was determined with the sulphuric acid anthrone method [51]. The vitamin C content in the plants was determined by the spectrophotometric procedure of Chanwitheesuk [52]. The sugar/acid ratio was calculated using the ratio of soluble sugar to titratable acid [53]. A comprehensive evaluation of tomato fruits subjected to different functional fertiliser treatments was conducted using the entropy weight TOPSIS model [54].

#### 4.4.4. Soil Fertility Indicators

Soil enzyme activity was analysed following standardised protocols from Guan [55]: urease via indophenol blue colorimetry (NH_3_-N release), alkaline phosphatase using a p-nitrophenyl phosphate (PNPP) substrate, sucrase through 3,5-dinitrosalicylic acid (DNS) reagent, and dehydrogenase via 2,3,5-triphenyltetrazolium chloride (TTC) reduction.

Soil pH was determined in a 1:5 (*w*/*v*) soil–water suspension using a calibrated pH meter (250A/610 model, Fisher Scientific, Waltham, MA, USA). Total nitrogen (TN) was quantified via the Kjeldahl method, followed by molybdenum blue colorimetric detection [56]. Total phosphorus (TP) content was determined spectrophotometrically (UV-2550, Shimadzu, Kyoto, Japan) following acid digestion [56]. Total potassium (TK) was extracted with sodium carbonate (Na_2_CO_3_) and quantified by flame photometry [57]. Soil organic matter (SOM) was measured via potassium dichromate (K_2_Cr_2_O_7_) oxidation and titration [58]. Available potassium (AK) was extracted with ammonium acetate, while the available phosphorus (AP) was determined by Olsen’s bicarbonate extraction [59]. Alkali-hydrolyzable nitrogen (AN) was measured by the alkaline diffusion method [60].

### 4.5. Statistical Analysis

Generalisation of experimental data and comprehensive evaluation by the entropy-weighted TOPSIS method were conducted using Excel v2021 (Microsoft Corp., Redmond, WA, USA). Mean values were compared for significant differences by Duncan’s test option in analysis of variance using SPSS v27.0 Statistics (IBM Corp., Armonk, NY, USA). The level of significance used in the tests was *p* ≤ 0.05. All plots were created using Origin v2024 (Origin Lab Corp., Northampton, MA, USA).

## 5. Conclusions

This study systematically analysed the effects of the synergistic interaction between functional fertiliser and vermicompost on the growth, fruit quality, and soil fertility of two tomato varieties. The principal findings revealed that the T1 treatment (mineral-sourced potassium fulvate fertiliser) demonstrated optimal efficacy in enhancing plant growth, increasing yield, and reducing the proportion of malformed fruits. In contrast, the T4 treatment (Type II algal polysaccharide fertiliser additive) exerted the most pronounced effect on improving comprehensive fruit quality indices. These results indicate that functional fertilisers can differentially modulate tomato growth and quality formation by regulating soil enzyme activity and nutrient bioavailability. This study provides a preliminary basis for fertiliser selection in similar ecological cultivation models by systematically comparing the effects of different functional fertilisers on a vermicompost-based substrate. Future research should focus on elucidating the underlying molecular mechanisms of this synergy, conducting multi-location and multi-season validation trials, and investigating a broader range of functional fertiliser types and application dosages to fully clarify their potential application in sustainable tomato production.

## Figures and Tables

**Table 1:** Effects of different functional fertilisers on soil physicochemical properties.

Variety	Treatment	SOM (g·kg^−1^)	pH	TN (g·kg^−1^)	TP (g·kg^−1^)	TK (g·kg^−1^)	AN (mg·kg^−1^)	AP (mg·kg^−1^)	AK (mg·kg^−1^)
DRK0568	CK	44.68 ± 1.00 b	7.98 ± 0.02 d	2.32 ± 0.20 b	2.08 ± 0.15 ab	19.47 ± 0.85 a	318.28 ± 2.85 b	95.54 ± 0.04 e	245.25 ± 1.25 d
	T1	53.09 ± 0.58 a	7.97 ± 0.02 d	2.86 ± 0.02 a	2.18 ± 0.15 a	20.45 ± 0.38 a	365.16 ± 11.58 a	98.27 ± 0.09 d	285.25 ± 0.25 a
	T2	52.42 ± 2.00 a	8.12 ± 0.02 a	2.89 ± 0.01 a	1.99 ± 0.08 ab	20.37 ± 0.45 a	366.79 ± 7.35 a	104.00 ± 0.09 a	238.50 ± 0.50 e
	T3	52.14 ± 1.00 a	8.06 ± 0.01 c	2.75 ± 0.06 a	1.89 ± 0.06 b	16.55 ± 1.14 b	358.18 ± 7.86 a	98.45 ± 0.09 c	240.50 ± 10 e
	T4	46.08 ± 2.00 b	8.08 ± 0.01 bc	2.81 ± 0.02 a	2.09 ± 0.01 ab	19.16 ± 1.00 a	345.41 ± 12.70 a	99.12 ± 0.04 b	264.00 ± 2.00 b
	T5	50.24 ± 0.09 a	8.09 ± 0.01 b	2.4 ± 0.01 b	2.20 ± 0.16 a	21.10 ± 0.15 a	321.83 ± 11.61 b	94.33 ± 0.09 f	252.50 ± 1.00 c
Sangfen 180	CK	38.90 ± 3.03 c	8.41 ± 0.02 b	1.19 ± 0.12 e	1.47 ± 0.02 f	16.99 ± 0.05 c	225.04 ± 4.36 f	87.35 ± 1.73 c	209.50 ± 3.77 d
	T1	46.25 ± 1.00 b	8.26 ± 0.02 c	2.33 ± 0.10 b	1.93 ± 0.02 b	14.43 ± 0.10 d	359.54 ± 2.66 a	95.16 ± 1.99 b	213.42 ± 5.51 d
	T2	43.78 ± 3.00 bc	8.21 ± 0.02 d	1.96 ± 0.14 c	1.86 ± 0.01 c	17.54 ± 0.10 b	335.36 ± 3.55 c	94.17 ± 3.10 b	240.75 ± 3.54 b
	T3	52.74 ± 0.91 a	8.23 ± 0.01 d	2.50 ± 0.01 a	1.79 ± 0.01 d	16.76 ± 0.10 c	344.89 ± 4.49 b	99.97 ± 0.09 a	223.25 ± 2.84 c
	T4	39.72 ± 4.00 c	8.5 ± 0.01 a	1.42 ± 0.06 d	1.57 ± 0.01 e	18.01 ± 0.49 a	258.19 ± 6.13 e	81.08 ± 1.05 d	253.33 ± 7.37 a
	T5	43.65 ± 0.92 bc	8.01 ± 0.01 e	2.26 ± 0.04 b	1.99 ± 0.01 a	18.40 ± 0.10 a	311.06 ± 5.98 d	99.59 ± 0.71 a	240.00 ± 1.5 b

Note: Soil organic matter (SOM), soil pH, total nitrogen (TN), total phosphorus (TP), total potassium (TK), alkali-hydrolyzable nitrogen (AN), available phosphorus (AP), and available potassium (AK). Data are expressed as mean ± standard deviation. Different letters within the same column indicate a significant difference at the 5% level.

**Table 2:** Effects of five functional fertilisers on soil enzyme activity.

Variety	Treatment	Soil Urease Activity (µg·d^−1^·g^−1^)	Soil Catalase Activity (µg·h^−1^·g^−1^)	Soil Sucrase Activity (mg·d^−1^·g^−1^)	Soil Alkaline Phosphatase Activity (µg·h^−1^·g^−1^)
DRK0568	CK	115.13 ± 3.24 c	1.32 ± 0.10 de	7.19 ± 0.13 c	71.58 ± 0.37 b
	T1	165.26 ± 4.69 b	1.51 ± 0.18 d	7.96 ± 0.05 a	77.75 ± 0.94 a
	T2	184.58 ± 5.95 a	3.42 ± 0.11 b	7.79 ± 0.06 b	76.16 ± 0.58 a
	T3	160.94 ± 6.11 b	5.45 ± 0.18 a	7.64 ± 0.06 b	76.98 ± 0.10 a
	T4	162.16 ± 1.76 b	1.10 ± 0.06 e	7.73 ± 0.10 b	77.23 ± 1.10 a
	T5	116.08 ± 0.80 c	1.93 ± 0.05 c	7.63 ± 0.07 b	72.63 ± 1.22 b
Sangfen 180	CK	113.01 ± 5.11 e	1.59 ± 0.02 e	7.57 ± 0.10 c	65.02 ± 0.10 e
	T1	114.72 ± 3.98 e	2.87 ± 0.07 d	7.91 ± 0.04 b	77.51 ± 1.10 b
	T2	173.35 ± 1.50 b	3.22 ± 0.07 c	7.81 ± 0.20 b	74.37 ± 0.66 c
	T3	153.40 ± 1.56 d	4.51 ± 0.15 a	7.83 ± 0.16 b	85.56 ± 0.79 a
	T4	184.07 ± 3.75 a	4.17 ± 0.11 b	7.87 ± 0.06 b	62.72 ± 1.59 f
	T5	160.32 ± 0.72 c	1.13 ± 0.12 f	8.17 ± 0.05 a	69.58 ± 0.13 d

Notes: Data are expressed as mean ± standard deviation. Different lowercase letters indicate significant differences in the level of the same condition under different treatments (*p* < 0.05).

**Table 3:** Production process-specific fertiliser usage and dosage.

Treatment	Name	Form	Dosage (Hectare)
CK	Water	-	-
T1	Mineral-sourced potassium fulvate fertiliser	Powder	15 kg
T2	Humic acid water-soluble fertiliser	Powder	30 kg
T3	Water-soluble NPK fertiliser with silicon	Agent	15 L
T4	Type II algal polysaccharide fertiliser additive	Agent	15 L
T5	Peptide-enriched fish protein organic liquid fertiliser	Agent	30 L

## Data Availability

The raw data underpinning the conclusions of this article is available from the authors, and interested individuals may obtain it by making a request.
